# Peer Review of “Information Technology Ambidexterity, Digital Dynamic Capability, and Knowledge Processes as Enablers of Patient Agility: Empirical Study”

**DOI:** 10.2196/34107

**Published:** 2021-12-06

**Authors:** Ibrahim Taiwo Adeleke

**Affiliations:** 1 Health Records Federal Medical Centre Bida Nigeria

**Keywords:** IT ambidexterity, dynamic capabilities, digital dynamic capability, knowledge processes, patient agility, hospitals, information sciences, information technology, digital health, health care, digital transformation, research models

*This is a peer-review report submitted for the paper “Information Technology Ambidexterity, Digital Dynamic Capability, and Knowledge Processes as Enablers of Patient Agility: Empirical Study”*

## Round 1 Review

### Specific Comments

#### Major Comments

1. Methods
  - Describe the study [1] settings
  - Move the highlighted (in the reviewed manuscript) content under Data Collection Procedures to a new subsection under subsection heading Study Population (see comments in the reviewed manuscript)
  - The highlighted content should be under a new subsection heading Study Design
  - Provide content on another two subsection headings:
    - Sampling Techniques
    - Sample Size
  - Separate content under Data Collection Procedure into two new subsection headings
    - Data Collection Tool and Procedure
    - Data Analysis and Management
  - Move Table 1 to Analyses & Results section
  - Provide content under two new subsection headings:
    - Inclusion and Exclusion Criteria
    - Ethics Considerations

### Minor Comments

1. Abstract
  - Do not begin a sentence with abbreviation of figure
  - Use past tense under Methods (eg, consider ‘used’ in lieu of ‘uses’)
  - See comments in the reviewed manuscript
2. Introduction
  - Use physicians in lieu of doctors
  - Use health care providers not other medical professionals
  - Keep in-text citation to the end of sentence
  - Add health information management professionals among the key stakeholders
  - Consider reducing the whole of section 2 (Theoretical Background) to 1-2 paragraphs and keep it within the Introduction section just before your study objective. This is to reduce readers’ boredom.
  - Compress the content under research models and hypotheses
3. Results
  - Make your findings more visible here
  - Make your writing more readable to known and unknown readers
4. Discussion
  - Plausible and insightful discussion but not a reflection of the content under the Results. Make the Results section more readable and meaningful to your audience.
5. Figure
  - Use Fig not Figure
6. Acknowledgement
  - It is scientifically necessary that you acknowledge the numerous (n=107) participants, who are the major stakeholders in your research.
7. Reference
  - List at least 3 authors before et al
  - Follow the Referencing Style consistently
8. Others
  - Use participants not respondents
